# Joint Datum–Form Decomposition for Photogrammetric Acceptance of Large Precision Assemblies

**DOI:** 10.3390/s26216761

**Published:** 2026-10-26

**Authors:** Yinggang Guo

**Affiliations:** Northwest Institute of Nuclear Technology, Xi’an 710024, China; fariel_gyg@163.com

**Keywords:** photogrammetry, error separation, assembly metrology, datum, repeated measurement, large-scale metrology

## Abstract

**Highlights:**

**What are the main findings?**

- An independent per-epoch 6-DoF datum overestimates the assembly RMS by ≈43% in Monte Carlo ($\sigma_{M}=6.0$ μm) and by ≈79% on real five-repeat data at $n_{c}=4$ controls (median ≈ 51%, 5–95% range ≈ 6–168% over random four-point layouts), whereas JDFD returns the full-set reference $\mathrm{RMS}(\hat{I})=11.92$ μm within numerical noise at every $n_{c}$ tested; the bias drops below 2% only when $n_{c}\approx 32$.
- Residual-similarity peeling reduces $\mathrm{RMS}(\hat{M})$ from 5.57 μm (common datum without peeling) to 3.36 μm—and from 3.91 μm to 3.36 μm against the full-set independent-datum baseline—giving an uncontaminated within-session repeatability estimate.

**What are the implications of the main findings?**

- Use JDFD—or any equivalent common-datum scheme with residual-similarity peeling—to avoid the sparse-control bias of independent per-epoch registration; the resulting assembly RMS is independent of the sparse-control budget drawn by IND and remains consistent with the full-set reference even when tooling provides only a few controls.
- Base the acceptance decision on the $n_{c}$-invariant $\mathrm{RMS}(\hat{I})$, treating $\mathrm{RMS}(\hat{M})$ as a secondary quality metric rather than the primary acceptance criterion.

**Abstract:**

Digital industrial photogrammetry is used for acceptance of large precision assemblies, but separating the time-invariant assembly form error from the epoch-varying measurement perturbation remains challenging. Registering each repeat independently to CAD with a six-degree-of-freedom (6-DoF) datum from only a few control points absorbs part of the form error, biasing the assembly RMS. This article proposes joint datum–form decomposition (JDFD), a generalised Procrustes analysis that shares one CAD-anchored common rigid across repeats and removes each epoch’s residual whole-body similarity. JDFD offers two advantages. First, its assembly RMS is independent of the sparse-control budget: an independent per-epoch 6-DoF overestimates the assembly RMS by ≈43% in Monte Carlo ($\sigma_{M}=6.0$ μm) and by ≈79% on real data at $n_{c}=4$ (median ≈ 51% over random layouts), whereas JDFD returns the full-set reference $\mathrm{RMS}(\hat{I})=11.92$ μm at every $n_{c}$ tested. Second, by removing residual similarity, JDFD yields an uncontaminated within-session perturbation $\mathrm{RMS}(\hat{M})=3.36$ μm, compared with 5.57 μm without peeling and 3.91 μm against the full-set independent-datum baseline. The resulting assembly RMS exceeds the 10 μm specification, so acceptance should rest on the $n_{c}$-invariant $\mathrm{RMS}(\hat{I})$.

## 1. Introduction

Acceptance of large precision assemblies—reflector panels, moulds, aircraft skins—against a micrometre-level root-mean-square (RMS) specification is limited as much by how the residual is interpreted as by the instrument itself [1,2,3]. Industrial photogrammetry is the practical field method on structures several metres across [4,5,6]. After a best-fit datum, the signed residual *H* at each target still mixes manufacturing and assembly that do not change while the part sits still with photogrammetric perturbation that changes from network to network [7]. Classical error-separation methods on machine tools do not transfer directly to a freeform assembly observed by an optical network [8,9,10].

A concrete use case is millimetre/micrometre-level surface acceptance of modular large reflectors and similar panelised precision assemblies inspected by industrial photogrammetry with dense coded targets and scale bars [3,4,5]. Large-astronomical-reflector surveys—the Five-hundred-meter Aperture Spherical Telescope (FAST) and the Giant Magellan Telescope (GMT)—adopt the same photogrammetric-network philosophy, with much sparser tooling-ball control. In such campaigns the vendor software typically re-fits an independent six-degree-of-freedom (6-DoF) datum at every epoch. When that 6-DoF is identified from the same targets that carry the assembly form—or from only a few tooling balls—net tilt and piston of the form error are partly absorbed into the nuisance parameters. The mean of *m* repeats is unbiased for a pointwise time-invariant form-error field if the per-epoch perturbation $M_{ij}$ has zero expectation over repeats [11]—a condition on the repeat ensemble, not on the inter-repeat time interval—but only after the epochs share one common datum and residual whole-body similarity has been stripped.

The motivation is practical. In large precision assembly, tooling constraints often limit the number of stiff datum controls: the instrument frame is established by a handful of scale bars, a metrology tower, or a sparse tooling-ball network. The practitioner must then decide whether to register every repeat independently to this sparse datum, or to pool information across repeats before forming the residual. Registering independently risks biasing the assembly RMS upward when the 6-DoF absorbs a non-trivial fraction of the form error; pooling via the mean target set requires a method that separates the shared form error from the per-epoch perturbation. To address this decision problem, this article presents joint datum–form decomposition (JDFD), a restricted generalised Procrustes analysis (GPA) tailored to photogrammetric acceptance. JDFD makes the pooling step explicit and quantifies, both in Monte Carlo and on real data, how large the independent-datum bias can become under sparse tooling.

JDFD consists of one common rigid from the mean target set to CAD, followed by peeling a residual similarity of each epoch about the time-average normal field. The article addresses two concrete engineering questions: when JDFD improves on independent per-epoch registration (IND), and what conclusions a small number *m* of repeats can support about the time-invariant assembly when the panel set is left unchanged between networks. Section 2 reviews datum definition, GPA, and classical error separation; Section 3 gives the observation model and algorithmic listing; Section 4 reports the sparse-control Monte Carlo test, the real-data sparse-control test, and the five-repeat campaign; Section 5 discusses the implications and limitations; Section 6 concludes.

## 2. Related Work

### 2.1. Datum Definition in Large-Scale Metrology

The datum is the operator that turns raw photogrammetric coordinates into a residual field suitable for engineering interpretation. In machine-tool metrology, the part coordinate system is fixed by the workpiece itself or by a fixture, so the datum is essentially given and only small rigid drifts remain to be absorbed by a 6-DoF best fit [8,10]. In photogrammetry of a freeform assembly, the datum is realised by a tooling-ball or scale-bar network and is not part of the part: the same network carries the assembly form-error field (the signed normal deviation of the assembled surface from CAD at every homologous target; we refer to this quantity throughout the article as the *form error*) [3,4,5]. Two consequences follow. First, when the same targets are used to define the datum and to evaluate the form error of the inspected assembly surface, the residual on the datum-defining subset is biased toward zero; this is the sparse-control failure mode that motivates the present article. Second, an independent per-epoch 6-DoF identified from $n_{c}$ controls absorbs a different fraction of the form error into the nuisance parameters at each epoch, because each epoch has its own measurement noise and its own measured control-point coordinates; as a result, the residuals $H_{ij}$ are not directly comparable across epochs.

Two practical conventions are used in vendor software (GOM, AICON, Metronor, and similar photogrammetry suites). Either the part is registered to CAD by an independent 6-DoF at every epoch using all targets, or a fixed subset of targets is reserved as controls and the remainder is treated as inspection points. The first convention averages the assembly into the rigid component at every epoch; the second exposes the failure mode that Section 4.3 quantifies. Free-network adjustment with inner constraints [4] removes the dependence on a chosen subset but cannot separate the form error from the per-epoch perturbation when both are carried by the same set of homologous targets. JDFD differs from free-network adjustment in that it does not solve for the network geometry itself: it takes as given the per-epoch target sets already reconstructed by the vendor software, and separates the shared CAD-anchored form error from the per-epoch similarity nuisance—which is the quantity needed for acceptance.

### 2.2. Generalised Procrustes Analysis

Generalised Procrustes analysis (GPA) aligns a set of homologous shapes by estimating a common mean shape and a similarity transformation for each shape [12]. In its original form, GPA deals with landmarks on objects that have no external reference: the mean is the Procrustes mean of the aligned shapes, and the transformations are restricted to translation, rotation and uniform scale [13]. Applications range from morphometrics [14] and medical-image registration to statistical shape analysis more broadly [15]. In GPA, the term *shape* refers to the geometric configuration of homologous landmarks—the spatial pattern of the points themselves—and the reference shape is unconstrained: it is whatever shape the data support.

Industrial acceptance is different. What is reported is not a free shape of the inspected part, but the *form error* field: the signed normal deviation of the assembled surface from CAD. Therefore, the reference shape in JDFD is not a free Procrustes mean, but the CAD-anchored normal graph. JDFD can be understood as a restricted GPA: the reference shape is fixed to the CAD nominal surface, and each epoch’s similarity is estimated in the tangent space of that reference shape. This restriction is what allows JDFD to output a CAD-anchored residual field—the form error.

### 2.3. Error Separation for Repeated Measurement

Classical error separation, reviewed by Whitehouse [8], Estler [9] and Evans et al. [10], exploits deterministic reversals or step tests to separate the straightness error of a machine from the probe error. These methods require a known mechanical reversal and a single sensitive axis. In photogrammetric acceptance, however, the inspected surface is freeform and is observed by an optical network, so no mechanical reversal is available. Classical error separation therefore does not transfer directly to this setting. The standard practice is instead to repeat the network *m* times and average pointwise, under the assumption that the per-epoch perturbation $M_{ij}$ has zero expectation over repeats [11]—again, a condition on the repeat ensemble, not on the inter-repeat interval. The repeat mean then recovers the time-invariant form error $I_{i}$, provided the epochs share one common datum and any residual whole-body similarity has been stripped. JDFD implements exactly that recipe in a single optimisation, with the reference shape fixed to the CAD nominal surface.

### 2.4. Positioning of This Work

Compared with an independent per-epoch 6-DoF (IND), JDFD replaces the per-epoch datum identification with a shared common-datum alignment followed by a per-epoch similarity peeling; compared with a free-network GPA, JDFD constrains the reference shape to the CAD-normal graph; compared with classical error separation, JDFD inherits the zero-mean averaging argument but adds the CAD anchoring required for acceptance decisions. The empirical claim of the article is that these three changes together are necessary to make the zero-mean averaging argument hold under sparse-control datums on a real industrial inspection. The Monte Carlo (Section 4.3.3) and the real-data sparse-control test (Section 4.3) are designed to expose and quantify this effect.

## 3. Methods

### 3.1. Overview of the Procedure

JDFD operates on *m* homologous target sets that are already expressed in a common measurement coordinate system. It has four stages:

1. Form the mean target set and estimate one common rigid transformation $\left(R_{0},t_{0}\right)$ from this mean set to the computer-aided design (CAD) surface (Horn absolute orientation [16]; no common scale).
2. Apply this common rigid transformation (Equation (1)) to every epoch and compute the signed normal residuals $H_{ij}$ (Equation (2)).
3. For each epoch, estimate and remove a residual similarity transformation $\left(\omega_{j},t_{j},\sigma_{j}\right)$ about the current time-average surface form error; then re-anchor the mean target set to CAD and iterate.
4. Report ${\hat{I}}_{i}=m^{-1}\sum_{j}H_{ij}$ (the estimated form error, a scalar field on the homologous targets) and ${\hat{M}}_{ij}=H_{ij}-{\hat{I}}_{i}$ (the estimated per-epoch perturbation field), i.e., the decomposition of Equation (3).

Stage 1 alone is the common-datum (CD) estimator. IND instead fits a new 6-DoF transformation from epoch *j* to CAD (often using $n_{c}$ controls) before averaging. Algorithm 1 summarises the iteration. Learned spatial regressors that map $\left(H_{i1},\ldots ,H_{im}\right)$ to $I_{i}$ were considered in exploratory work but are not used here: training such a regressor by minimising the pointwise mean-square error tends to underestimate $\mathrm{RMS}(\hat{I})$, which is unsuitable for acceptance testing against a fixed specification.

### 3.2. Observation Model Under a Common Datum

Let *n* homologous targets have CAD coordinates $C_{i}\in R^{3}$ and outward unit normals $n_{i}$. For a shallow dish, we take $n_{i}$ as the third principal direction of $\left\{C_{i}\right\}$. For a general body, per-target CAD normals can be used; a highly folded surface may require local normals.

Epoch *j* yields measured coordinates $P_{ij}$ in the instrument frame. A single rigid transformation maps every epoch into the CAD frame:

(1) $$Q_{ij}=R_{0}P_{ij}+t_{0},$$

and the signed normal residual is

(2) $$H_{ij}=n_{i}^{⊤}\left(Q_{ij}-C_{i}\right).$$

We decompose the residual as

(3) $$H_{ij}=I_{i}+M_{ij},$$

where $I_{i}$ is the time-invariant form error and $M_{ij}$ is the per-epoch perturbation. We assume $E_{j}\left[M_{ij}\right]=0$ at each target: the form error does not change systematically from one repeat to another, regardless of the time interval between networks. Any bias that is identical in every network is absorbed into $I_{i}$ and cannot be separated from it using repeats alone.
**Algorithm 1** Joint datum–form decomposition (JDFD)**Require:** Homologous target coordinates $P_{ij}$, CAD coordinates $C_{i}$, normals $n_{i}$; epochs j=1,…,m  1:${\bar{P}}_{i}\leftarrow m^{-1}\sum_{j}P_{ij}$; estimate the rigid transformation $\left(R_{0},t_{0}\right)$ from $\left\{{\bar{P}}_{i}\right\}$ to $\left\{C_{i}\right\}$ [16]  2:$Q_{ij}\leftarrow R_{0}P_{ij}+t_{0}$; $H_{ij}\leftarrow n_{i}^{⊤}\left(Q_{ij}-C_{i}\right)$  3:**for** k=1 to *K* **do** ▹ iterate until the change in $\mathrm{RMS}(\hat{I})$ between successive iterations falls below $10^{-3}$ μm, or K=6 iterations (both reached in all tests below)  4:       $I_{i}^{\left(k\right)}\leftarrow m^{-1}\sum_{j}H_{ij}$  5:       **for** j=1 to *m* **do**  6:           From the linearised residual twist of Equation (4), solve the least-squares problem in Equation (5) for $\left(\omega_{j},t_{j},\sigma_{j}\right)$  7:            Apply the estimated similarity transformation to $\left\{Q_{ij}\right\}$ and recompute $H_{ij}$  8:       **end for**  9:     Recompute the mean target set from the updated $\left\{Q_{ij}\right\}$; re-estimate $\left(R_{0},t_{0}\right)$ to CAD; remap all epochs 10:**end for** 11:**return** ${\hat{I}}_{i}\leftarrow m^{-1}\sum_{j}H_{ij}$, ${\hat{M}}_{ij}\leftarrow H_{ij}-{\hat{I}}_{i}$

RMS values are quadratic means over the discrete surface form-error field [7,17]. Throughout this article, $\mathrm{RMS}(\hat{I})$ denotes the RMS form error of the estimated assembly surface, and $\mathrm{RMS}(\hat{M})$ denotes the perturbation RMS—the RMS of the estimated per-epoch perturbation field. Throughout, *per-epoch perturbation* refers to the residual field $M_{ij}$ of Equation (3), and *per-epoch similarity* to the similarity parameters $\left(\omega_{j},t_{j},\sigma_{j}\right)$ that JDFD peels; the two are not interchangeable. Writing ${\bar{H}}_{i\;\cdot \;}=m^{-1}\sum_{j}H_{ij}$ and $M_{ij}=H_{ij}-{\bar{H}}_{i\;\cdot \;}$, the identity $\mathrm{RMS}{\left(H\right)}^{2}=\mathrm{RMS}{\left(\bar{H}\right)}^{2}+\mathrm{RMS}{\left(M\right)}^{2}$ holds algebraically.

### 3.3. Independent Datum Versus Residual Similarity

Software reports typically re-fit $\left(R_{j},t_{j}\right)$ from epoch *j* to CAD. Even a sub-arcsecond residual rotation produces a normal-direction displacement that grows linearly with the lever arm, so its contribution can become comparable to the form error being measured. IND therefore mixes the quantity under test into the nuisance parameters. Leaving residual similarity in $H_{ij}$ (CD without peeling) leaves $\hat{I}$ unbiased under the zero-mean assumption but inflates RMS(M) by whole-body jitter.

### 3.4. Linearised Residual Twist

After Equation (1), a small rotation, translation and isotropic scale of epoch *j* change the signed residual by

(4) $$\delta H_{ij}={\left(Q_{ij}\times n_{i}\right)}^{⊤}\omega_{j}+n_{i}^{⊤}t_{j}+\sigma_{j}\;n_{i}^{⊤}Q_{ij}.$$

At iteration *k*, each epoch estimates these seven parameters by least squares:

(5) $$\min_{\omega_{j},\;t_{j},\;\sigma_{j}}\sum_{i=1}^{n}{\left(H_{ij}^{\left(k\right)}+\delta H_{ij}-I_{i}^{\left(k\right)}\right)}^{2}.$$

In other words, each epoch finds the overall rotation, translation and scale that best align its residual to the current mean form error. The estimated transformation is then applied, and the common rigid is refreshed so that these per-epoch transformations cannot absorb any net rigid component of the form error. The linearised model in Equations (4) and (5) treats the per-epoch residual as Gaussian white noise plus the smooth assembly field, so it has no explicit mechanism for local outliers. In practice, homologous-target matching and a per-target robust screen are applied before JDFD to reject gross mismatches; a residual that escapes this screen would be absorbed into $\hat{I}$ or the peeled similarity rather than flagged, so extending the peel to a robust (*M*-estimator) loss is a natural future extension.

The seven parameters $\left(\omega_{j},t_{j},\sigma_{j}\right)$ are *nuisance parameters*: they describe the overall pose and scale of epoch *j*, not the surface form error. Removing them allows the residual to be interpreted as an engineering form error. This step is equivalent to estimating a generalised Procrustes analysis (GPA) similarity [12], with the reference shape fixed to the CAD-normal graph $X_{i}=C_{i}+{\hat{I}}_{i}n_{i}$. In standard GPA the reference shape is free; here it is the CAD nominal surface plus the estimated form-error field ${\hat{I}}_{i}n_{i}$—a CAD-anchored form-error graph. This is why JDFD is a *restricted GPA*; see Section 2.2 for the relationship to standard GPA.

An unbiased $\mathrm{RMS}(\hat{M})$ applies the m−1 correction

$$\bar{\mathrm{RMS}}\left(\hat{M}\right)\;=\;\sqrt{\frac{m}{m-1}}\;\mathrm{RMS}\left(\hat{M}\right),$$

where $\mathrm{RMS}(\hat{M})$ is the sample (period-*m*) RMS of the perturbation field and $\bar{\mathrm{RMS}}\left(\hat{M}\right)$ is the corresponding unbiased estimator of the underlying RMS(M).

## 4. Results

### 4.1. Measurement Object and Experimental Setup

**Figure 1:**
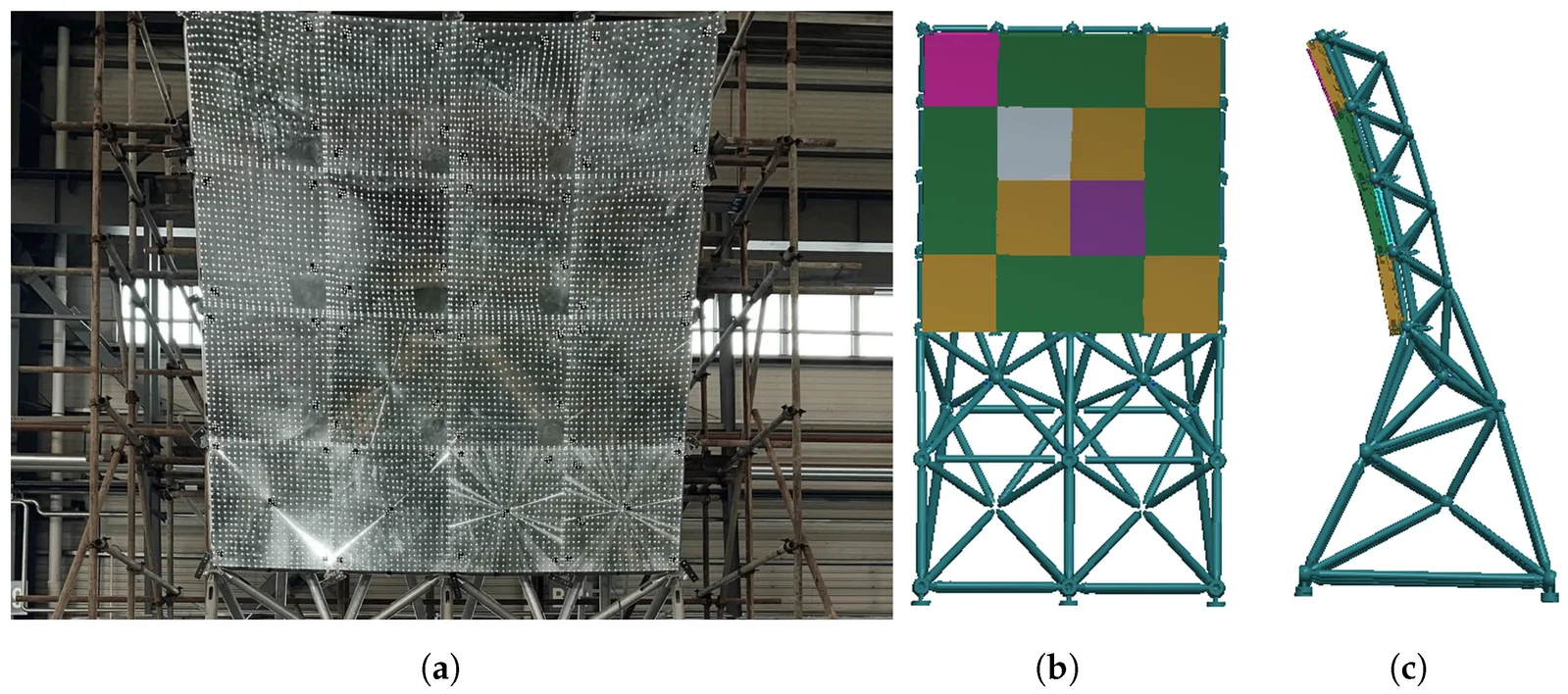
Measurement object and photogrammetric survey setup. (**a**) Indoor survey of the 3m-class terahertz reflector: panelised main reflector on the support truss, with coded targets; (**b**) CAD front view: 4×4 panel mosaic on the aperture; (**c**) CAD side view.

The five-repeat measurement campaign analysed below was acquired on a 3m-class terahertz reflector antenna (Figure 1). Its main reflector is a 3m×3m shallow paraboloidal surface assembled from a 4×4 array of aluminium panels on a ball-node truss skeleton, with fine-tuning mechanisms providing micrometre-level normal adjustment. The surface was surveyed in a single indoor session using a digital industrial photogrammetry system with dense homologous targets and scale bars [4]. The dataset consists of five independent networks *R*_1_–*R*_5_ aligned to a single CAD master, with 7182 homologous targets distributed over the panelised surface. Extra similarities from each epoch to CAD reached 0.0020° in rotation and 0.046 mm in *Z*, establishing the per-epoch perturbation regime on which Section 4.3 below quantifies the sparse-control failure mode.

### 4.2. Full-Set Decomposition and the Role of Residual-Similarity Peeling

Table 1 compares Vendor (independent CAD fits, then mean of reported totals), IND, CD and JDFD on the full n=7182 homologous set. Here IND denotes independent per-epoch 6-DoF registration using the full target set ($n_{c}=n=7182$); the sparse-control variant IND$\left(n_{c}\right)$ is introduced in Section 4.3. This subsection establishes the full-set JDFD reference $\mathrm{RMS}(\hat{I})=11.92$ μm. With n=7182, IND/CD/JDFD agree on $\mathrm{RMS}(\hat{I})$ to 0.02 μm: the per-epoch 6-DoF is stiff on the full target set, so the sparse-control failure mode reported in Section 4.3.3 below is avoided. Section 4.3 below confirms this on the real five-repeat data by varying $n_{c}$ over {4,8,16,32,64,128}.

The decisive change across estimators is the per-epoch perturbation field, not the form error. CD, which does not peel residual similarity, gives $\mathrm{RMS}(\hat{M})=5.57$ μm; JDFD yields 3.36 μm, with the corresponding unbiased estimator $\bar{\mathrm{RMS}}\left(\hat{M}\right)=3.76$ μm. Vendor’s $\mathrm{RMS}(\hat{M})=3.67$ μm and IND’s 3.91 μm differ by 0.24 μm (7% of the JDFD value), a difference of the size expected between two software pipelines reported by different convergence criteria rather than by a different datum choice: both use the full target set, as does the IND column of this table.

The assembly fails the acceptance threshold. The RMS of every epoch, and of $\hat{I}$, exceeds the 10 μm specification. Using the vendor local normals instead of the mean normal changes $\mathrm{RMS}(\hat{I})$ by only 0.02 μm, so the conclusion is robust to the normal definition.

Bootstrap and leave-one-out confirm that the point estimate is stable. Leave-one-epoch-out recomputation of JDFD gives 11.79–12.13 μm. A spatial block bootstrap on 400mm tiles (64 occupied blocks; the tile length was chosen *a priori* as the simulation GP correlation length of 400mm, before the real-data analysis) with 800 draws yields a 95% interval [11.28,12.61] μm on $\mathrm{RMS}(\hat{I})$ [18]; 250mm tiles (156 blocks) and 800mm tiles (16 blocks), computed with the same seed and the same 800 draws, give [11.40,12.45] and [11.09,12.76] μm [19]. The interval widens as the tiles grow, as expected for a field with structure on the panel scale.

The Vendor column is included for reference only. Unlike the other estimators, it follows the vendor’s own per-epoch datum choice, so its values are not directly comparable with IND, CD or JDFD. It uses the signed total residual reported by the vendor software for each epoch and computes the corresponding RMS values; a full per-target residual field was not available from the vendor software. The per-epoch vendor similarity parameters and the temporal-drift audit are given in Section 4.4. That audit shows no significant drift in $\mathrm{RMS}(H_{j})$, supporting the zero-mean assumption behind $\hat{I}={\bar{H}}_{i\;\cdot \;}$.

**Figure 2:**
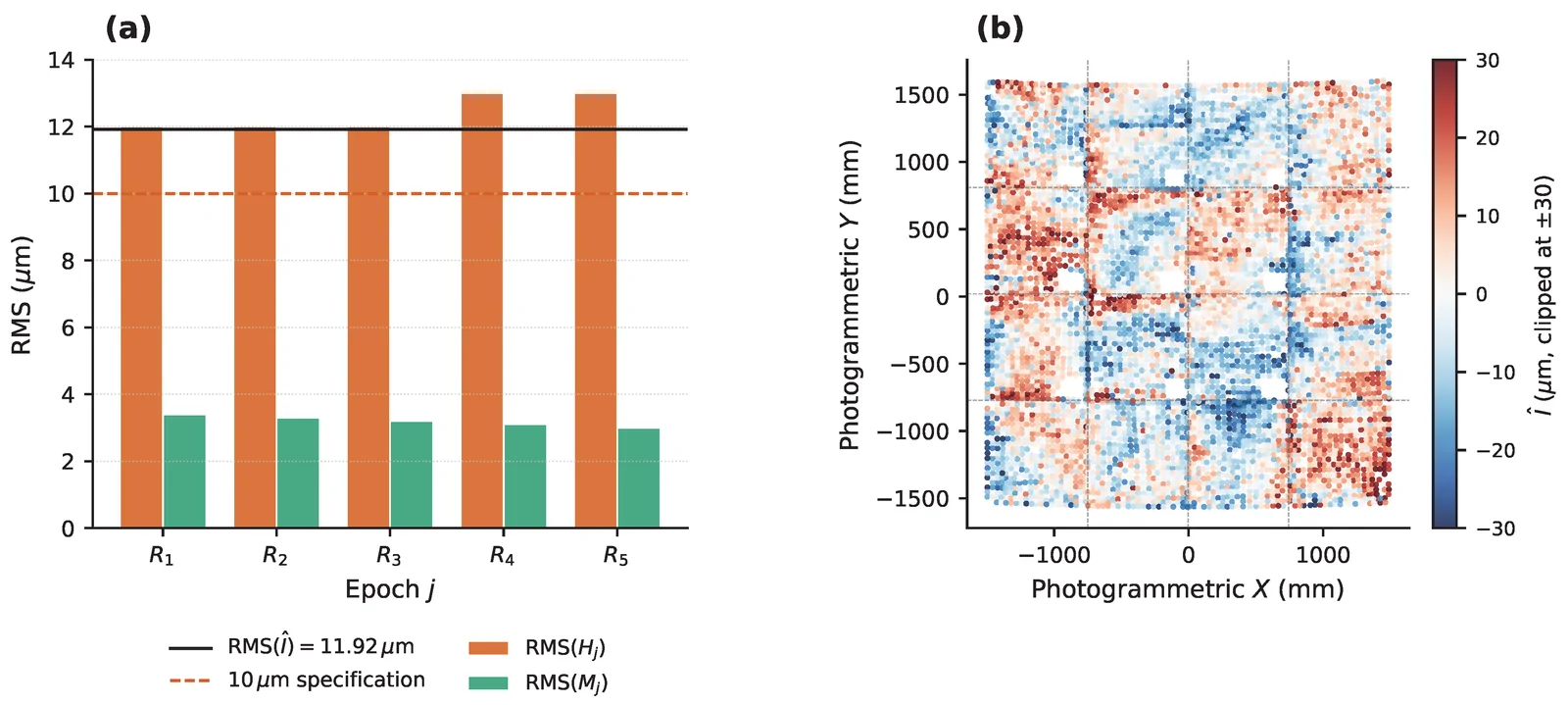
JDFD on the five repeats. (**a**) Per-epoch decomposition: orange bars, $\mathrm{RMS}(H_{j})$; green bars, $\mathrm{RMS}(M_{j})$; solid line, $\mathrm{RMS}(\hat{I})=11.92\;\mu m$; dashed line, the 10μm specification. (**b**) Spatial map of $\hat{I}$ after JDFD in the photogrammetric XY plane; colour bar in micrometres, clipped at ±30μm; the overlay marks the 4×4 panel mosaic on the 3×3m aperture. Panel-scale organisation is consistent with assembly rather than white measurement noise.

The per-epoch decomposition and the spatial organisation of the estimated form error $\hat{I}$ are visualised in Figure 2.

### 4.3. Sparse-Control Test on the Real Five-Repeat Data

The previous subsection established the full-set JDFD reference $\mathrm{RMS}(\hat{I})=11.92$ μm at $n_{c}=n=7182$. This subsection performs the same test on the five real homologous networks under deliberately sparse control budgets ($n_{c}\in \left\{4,8,16,32,64,128\right\}$), to verify that the sparse-control failure mode of IND is observable on real industrial data and that JDFD’s peeling step absorbs a deliberately poor initial pose. The Monte Carlo counterpart is derived in Section 4.3.3, and Section 4.3.5 explains why the real-data bias exceeds it.

#### 4.3.1. Test Design

For each $n_{c}$, we run two estimators on the same five homologous epochs, both compared against the full-set JDFD reference (Table 1):

- **IND**$\left(n_{c}\right)$ — per-epoch 6-DoF identified from exactly $n_{c}$ controls.
- **JDFD**$\left(n_{c}\right)$ — a three-stage procedure with a deliberately poor initialisation. *Stage A* estimates the common rigid transformation $\left(R_{0},t_{0}\right)$ from the full mean target set to CAD; the $n_{c}$ controls never enter the datum. *Stage B* initialises every epoch at the per-epoch rigid identified from the same $n_{c}$ controls—the pose a practitioner would obtain from sparse tooling, carrying a known sparse-control bias. *Stage C* runs the standard residual-similarity peeling loop (Algorithm 1, steps 2–3), which removes each epoch’s residual similarity and re-anchors the mean target set to the common datum at every iteration. The test is whether Stage C absorbs the bias introduced in Stage B and converges to the full-set reference.

Controls follow the same lattice rule as in Section 4.3.3: $k_{i}=\mathrm{round}\;\left(i\;\left(n-1\right)/\left(n_{c}-1\right)\right)$ for $i=0,\ldots ,n_{c}-1$. This matches the practical case where the tooling-ball layout is fixed before the campaign; for n=7182 and $n_{c}=4$, it gives $k_{0}=0$, $k_{1}=2394$, $k_{2}=4787$, $k_{3}=7181$.

A notation that is easy to misread: in JDFD$\left(n_{c}\right)$, the $n_{c}$ controls are used only to provide a deliberately poor initial pose; the common datum itself is always estimated from the full mean target set, as in the standard JDFD. This reflects the practical situation where a dense point cloud is available but only a few control points are used for per-epoch registration. The subscript $n_{c}$ therefore labels the *initialisation* supplied to JDFD, not the datum it uses.

#### 4.3.2. Results of the Sparse-Control Test

In Stage A, the common datum is identified from the full mean target set, so no $n_{c}$-controlled pose defines the datum; in Stage C, the peeling loop re-anchors the mean target set to CAD at every iteration and absorbs any residual per-epoch similarity into $\left(\omega_{j},t_{j},\sigma_{j}\right)$. As a result, the converged residuals $H_{ij}$ depend only on the common-datum mean and the peeling system; the only component that survives into $\hat{I}$ is the time-invariant form error $I^{⋆}$. The same reasoning applies to ${\hat{M}}_{ij}=H_{ij}-{\bar{H}}_{i}$, because the converged $H_{ij}$ no longer contains the $n_{c}$-control pose introduced in Stage B.

The invariance is in fact a first-order identity, not an empirical coincidence, and can be stated in one line. Peeling estimates a seven-parameter similarity $\left(\omega_{j},t_{j},\sigma_{j}\right)$ by regressing the residual field on the columns $p\times n_{i}$, $n_{i}$ and $p\;\cdot \;n_{i}$—functions that are linear (or constant) in the target position. A per-epoch rigid with a small rotation is the leading term of a similarity, so its induced height field lies in the span of those regressors. The rigid that Stage B fits from $n_{c}$ controls is a rigid, and a rigid fitted at $n_{c}$ targets is still a rigid, so its height field is in the same span and is removed by the very next peel; to first order the two cancel, regardless of how poor the initial pose is. Every JDFD$\left(n_{c}\right)$ run therefore returns $\mathrm{RMS}(\hat{I})=11.92\;\mu m$ to within $10^{-5}\%$ of the full-set reference (Table 2), which is a consistency check of the implementation rather than the statement itself. The argument is a linearisation and is expected to fail once the epoch pose stops being small against the aperture: injecting an artificial per-epoch rigid of $2^{\circ }$ rotation and 20 mm translation—orders of magnitude beyond the measured 7.2″ and 46 μm of the per-epoch similarity audit (Section 4.4.1)—raises $\mathrm{RMS}(\hat{I})$ by +358%, and a $40^{\circ }$/400mm pose by $+2\times 10^{5}\%$. Within the measured perturbation regime the invariance holds, and outside it the estimator is simply not being asked what it claims to deliver.

**Figure 3:**
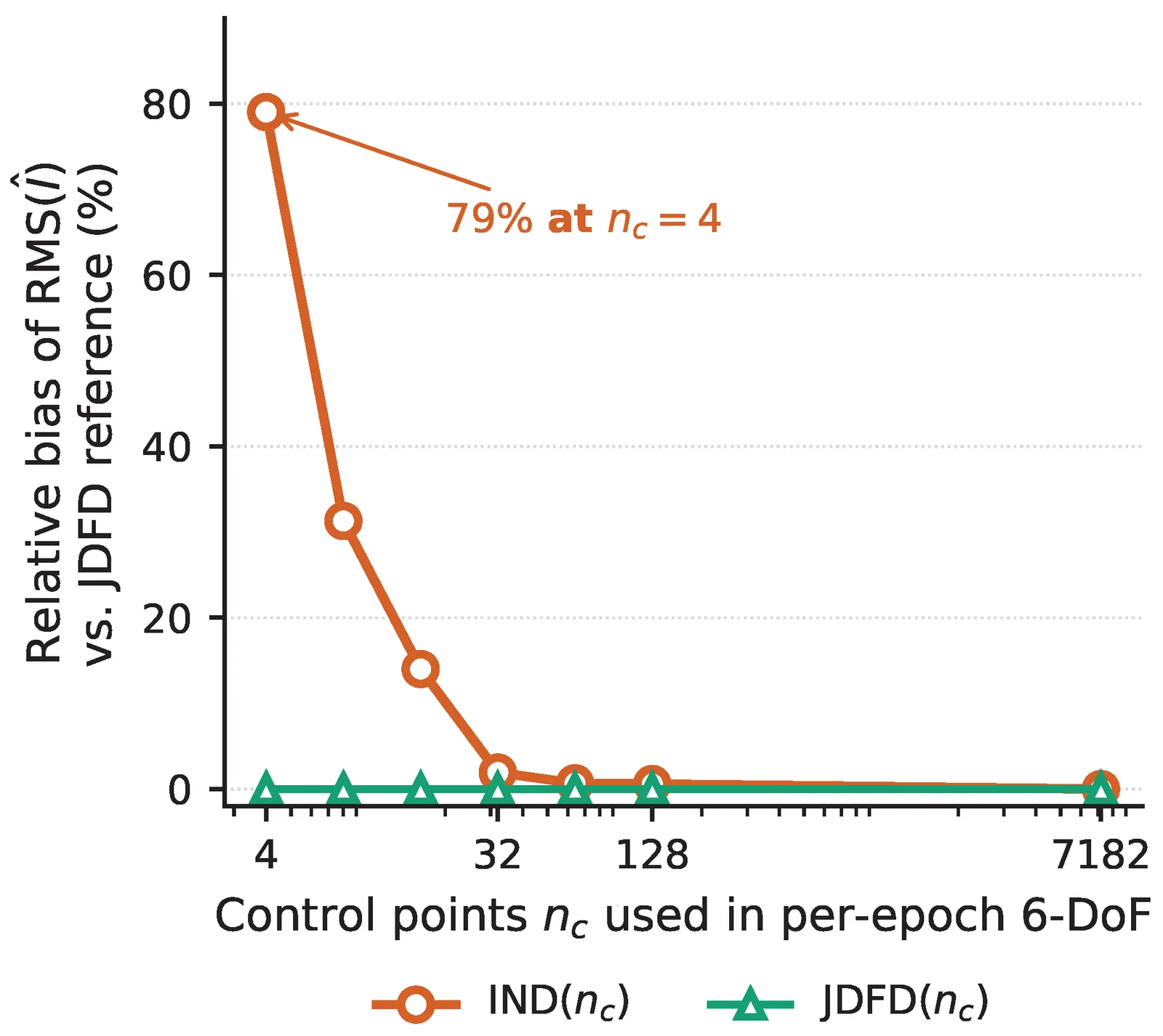
Sparse-control test on the real five-repeat data (n=7182, m=5). Relative bias of $\mathrm{RMS}(\hat{I})$ versus the full-set JDFD reference 11.92μm, as a function of the number of controls $n_{c}$. Circles: IND$\left(n_{c}\right)$; triangles: JDFD$\left(n_{c}\right)$ ($n_{c}$-control initialisation + peeling). At $n_{c}=4$, IND overestimates $\mathrm{RMS}(\hat{I})$ by≈79%, whereas JDFD returns the reference value within numerical noise. The JDFD markers are flat by construction (see Section 4.3.2); the IND curve is a single evenly index-spaced draw, and its instability under different layouts is quantified in Section 4.3.5.

With this invariance established, the real-data test reduces to a one-line comparison: IND$\left(n_{c}\right)$ versus the full-set reference 11.92μm. Figure 3 plots the relative deviation $\left|\mathrm{RMS}(\hat{I})-11.92\right|/11.92$ (in %) and Table 2 lists the numerical values (the full sweep in machine-readable form in Table S3 and Data S3).

At $n_{c}=4$, the real-data IND overestimates $\mathrm{RMS}(\hat{I})$ by ≈79%, ≈2× the 39.9% predicted by the matched-amplitude Monte Carlo of Section 4.3.4 (the headline $\sigma_{M}=6\;\mu m$ Monte Carlo gives 42.6%). The gap closes only when $n_{c}\approx 32$ (relative bias <2%). JDFD returns the reference value within numerical noise at every $n_{c}$ tested.

A note on what these real-data numbers do and do not establish follows. On the five delivered networks the only thing that varies is $n_{c}$, and what is measured is therefore the dependence of each estimator on the control budget: IND moves; JDFD does not. That the sparse-control figure is an overestimate, rather than an underestimate, cannot be read off the real data, because no ground truth is available there; the upward direction is established in simulation (Section 4.3.3 and Section 4.3.6) and is reproduced here with the measured field in the role of the truth. The real-data figures are also one draw of the control layout, and Section 4.3.5 quantifies how far that draw sits from the centre of its own distribution.

#### 4.3.3. Monte Carlo Prediction of the Sparse-Control Bias

**Figure 4:**
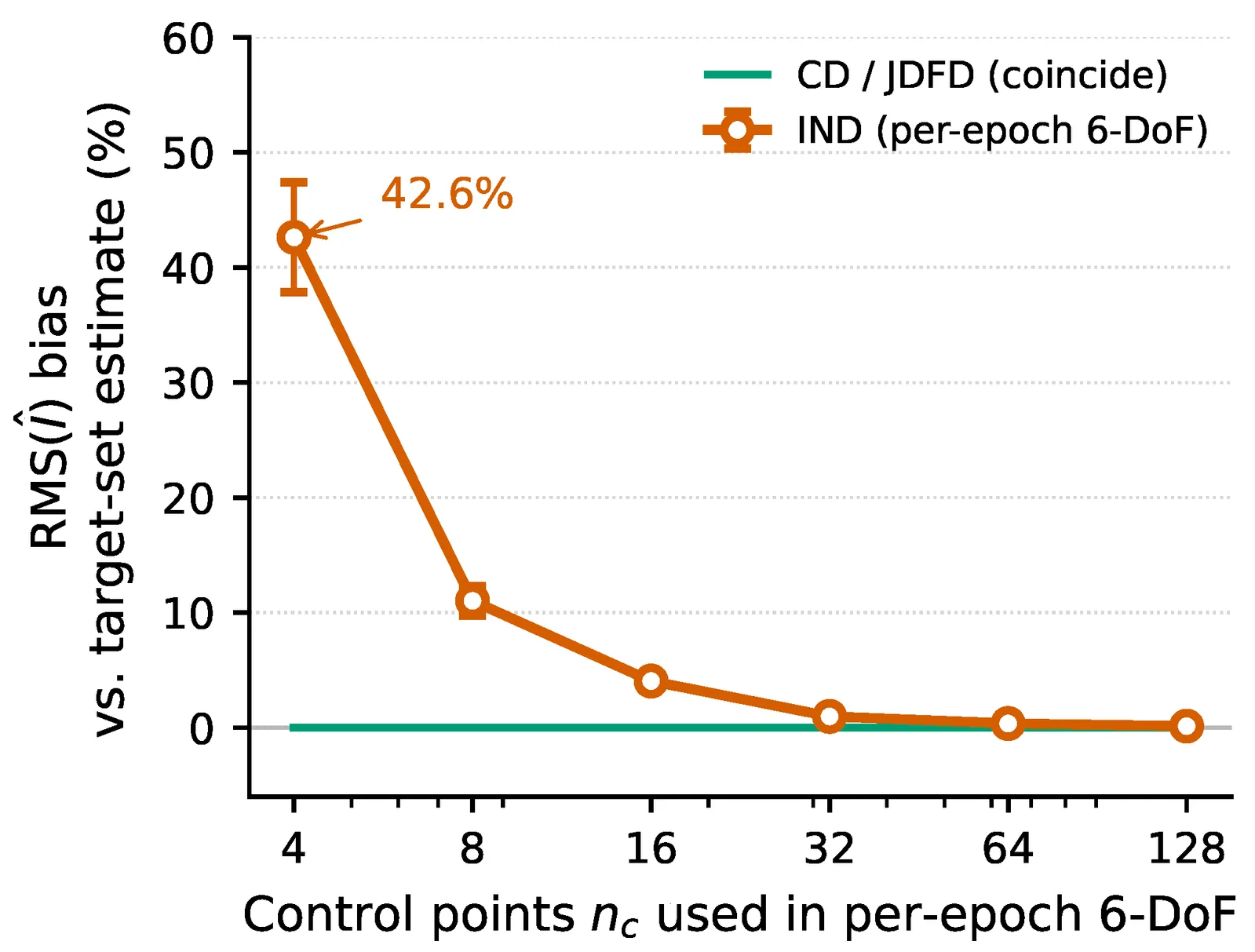
Bias of $\mathrm{RMS}(\hat{I})$ relative to the CD/JDFD target-set estimate versus the number of control points $n_{c}$ used by IND; markers are means over 200 draws with 95% CIs. IND is inflated by 42.6% at $n_{c}=4$ and falls below 1% only at $n_{c}=32$, whereas CD and JDFD, which register the mean target set and so are independent of $n_{c}$, are zero by construction. Simulation: true RMS(*I*) = 12μm, $\sigma_{M}=6\;\mu m$, m=5.

To isolate the mechanism behind the real-data bias, this subsubsection uses a controlled Monte Carlo on the same CAD. A shallow paraboloidal dish of aperture 3m is sampled on a 24×24 grid. The true assembly $I^{⋆}$ is a Gaussian-process field (correlation length 400mm) mixed with white machining noise and scaled to RMS 12μm; photogrammetric noise is white with $\sigma_{M}=6\;\mu m$. Each epoch receives a random similarity transformation, with translation, rotation and scale components having standard deviations of 40μm, 8″, and 20ppm, respectively. IND uses $n_{c}$ control points selected on a lattice; CD and JDFD register the mean target set. Figure 4 plots the bias of $\mathrm{RMS}(\hat{I})$ against the CD/JDFD target-set estimate on the same draw—the convention already used for the real data in Figure 3—over 200 independent draws per cell, with 95% confidence intervals of the mean. With 4 control points, IND overestimates $\mathrm{RMS}(\hat{I})$ by 42.6% (95% CI [37.8,47.4]%); the bias falls to 4.1% at $n_{c}=16$ and below 1% only at $n_{c}=32$. CD and JDFD register the mean target set, so the $n_{c}$ controls do not enter their estimate and their bias is zero by construction. The IND gap closes only when several dozen control points are supplied—precisely when the datum is not realised from only a handful of control points. Pointwise recovery $\mathrm{RMS}(\hat{I}-I^{⋆})$ is already ≈4.7 μm at m=5 for CD/JDFD and improves only slowly toward m=12. Thus the decisive advantage of JDFD over IND is sparse-control bias protection, not a large further gain once the 6-DoF is stiff on thousands of targets.

#### 4.3.4. Sensitivity to the Perturbation Amplitude

**Figure 5:**
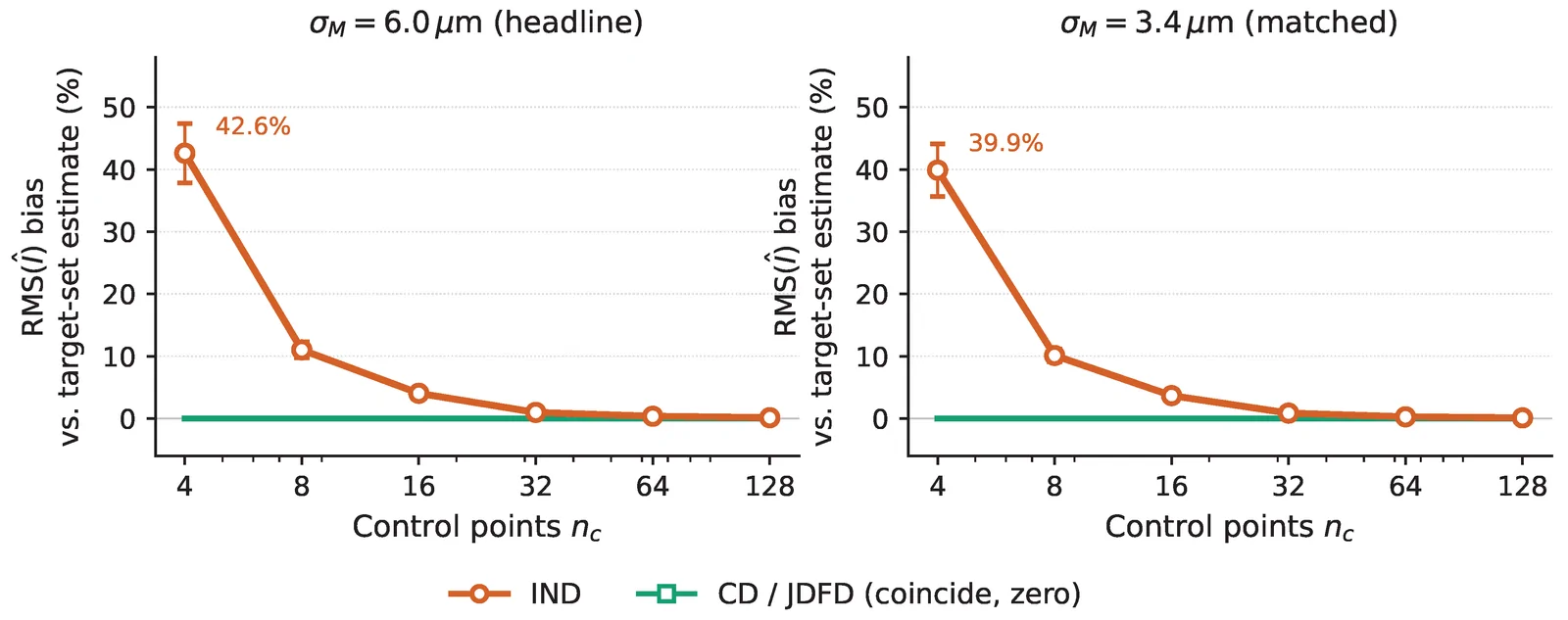
Sensitivity of the sparse-control bias to the perturbation amplitude. Bias of $\mathrm{RMS}(\hat{I})$ relative to the CD/JDFD target-set estimate; markers are means over 200 draws, whiskers are 95% confidence intervals of the mean. At $n_{c}=4$, the IND bias is 39.9% under the matched-amplitude $\sigma_{M}=3.4\;\mu m$ and 42.6% at the headline $\sigma_{M}=6.0\;\mu m$; the two intervals overlap substantially and both fall below 1% by $n_{c}=32$. CD and JDFD coincide at zero, with the JDFD-only benefit on $\mathrm{RMS}(\hat{M})$ (Table 1).

The headline simulation uses $\sigma_{M}=6\;\mu m$, a conservative upper bound relative to the measured unbiased $\mathrm{RMS}(\hat{M})=3.76\;\mu m$ reported in Section 4.2. To test whether the conclusion depends on this choice, Figure 5 repeats the sparse-control sweep at $\sigma_{M}=3.4\;\mu m$ (matched to the real perturbation amplitude) and at $\sigma_{M}=6.0\;\mu m$, with 200 draws per cell. The qualitative conclusion is unchanged: at $n_{c}=4$, the IND bias is 39.9% (95% CI [35.7,44.1]%) under the matched-amplitude noise and 42.6% ([37.8,47.4]%) at the headline amplitude, and falls below 1% by $n_{c}=32$ in both cases. CD and JDFD remain at zero because both register the mean target set; the JDFD-only advantage remains on $\mathrm{RMS}(\hat{M})$ (Table 1). The sparse-control failure mode of IND is therefore not an artefact of an over-conservative $\sigma_{M}$: the two amplitudes give statistically indistinguishable biases, their confidence intervals overlapping over most of their range.

#### 4.3.5. Why the Real-Data Bias Exceeds the Prediction

The real-data IND bias at $n_{c}=4$ (79%) exceeds the 42.6% predicted on the synthetic paraboloid in Section 4.3.3. Three candidate explanations can be tested directly on the real CAD, and only two survive.

**Perturbation model.** Replacing white noise by a systematic per-epoch similarity matched to the measured amplitude leaves the IND(4) bias unchanged (29.3% vs. 32.1% with white noise, CIs overlapping; full cell output in Table S2 and Data S1). The perturbation model is therefore immaterial.

**Spatial content of the assembly field.** The real form error is panel-scale, whereas the simulated field carries a single 400mm length scale. Removing the panel-scale content of the measured field lowers the predicted IND(4) bias from 84.2% to 50.1% (Section 4.3.6), so the spatial content of the assembly field accounts for roughly two-fifths of the discrepancy.

**Control-point placement.** The evenly index-spaced subset used above is an unfavourable draw. Over 200 random four-point subsets the median IND bias is 51%, with 174 non-degenerate draws spanning 0.8–198% (Table S4, Data S2). The measured 79% sits at the 70th percentile: it is representative of the upper half rather than of its centre.

The matched-amplitude Monte Carlo is therefore not misleading, but by itself it is insufficient to predict the real-data failure mode.

#### 4.3.6. Monte Carlo on the Measured Assembly Field

The synthetic carrier of Section 4.3.3 is a smooth paraboloid carrying a 400mm Gaussian-process field. The measured assembly field is neither smooth nor isotropic, and Section 4.3.5 asks whether that difference, rather than the control layout alone, is what drives the real-data bias. The Monte Carlo is therefore repeated with the measured field itself as the truth.

The epochs are $C_{i}+n_{i}\left(I_{i}^{⋆}+M_{ij}\right)$ with $I^{⋆}$ the published field of Data S5 (RMS=11.92μm, 7182 targets) and $M_{ij}$ white at $\sigma_{M}$; the control lattice rule, the seed, the 200 draws per cell and the Student-*t* intervals are those of Section 4.3.3. No epoch similarity jitter is included, because the vendor absorbs the per-epoch similarity before delivery (Section 4.4.1): putting it back unabsorbed would place the common-datum $\mathrm{RMS}(\hat{M})$ near 55μm, where the measured value is 5.57μm. The field is held fixed across draws, so Data S5 is used as a pseudo-truth, not as an independent ground truth; it is the article’s own estimate, and it is the field against which the acceptance decision is actually made.

Under that model the predicted IND curve reproduces the measured one at every $n_{c}$ (Table 2 gives the measured column; Table S5 and Data S6 give the simulated one). At $\sigma_{M}=6\;\mu m$ and $n_{c}=4,8,16,32,64$, the simulated $\mathrm{RMS}(\hat{I})$ is 22.50,15.67,13.84,12.47 and 12.31μm against the measured 21.34,15.65,13.59,12.15 and 12.00μm; the discrepancy never exceeds 1.2μm, and the shape of the decay—a large bias at $n_{c}=4$ collapsing below 2% by $n_{c}=32$—is reproduced without any tuning of the field. In the truth convention, which no figure of this article has used so far, IND(4) is +88.8% (95% CI [87.2,90.3]%) and CD and JDFD are both +2.5%; under the matched amplitude $\sigma_{M}=3.4\;\mu m$ the same figures are +87.6% and +0.8%.

Two readings follow. First, the real-data sparse-control bias is a property of the estimator acting on the measured field and is reproduced end to end. Second, it is independent of the peeling step: in this configuration CD and JDFD return the same $\mathrm{RMS}(\hat{M})$, 5.37μm at $\sigma_{M}=6\;\mu m$ and 3.04μm at 3.4μm, because the model contains no whole-body similarity for peeling to remove. The two advantages claimed for JDFD are therefore separable—the sparse-datum bias is reproduced here, the peeling gain is not, and the gain rests on the delivered clouds of Section 4.2 rather than on the estimator’s behaviour under white noise.

Whether the panel-scale content is the cause is then tested directly, by swapping the field and keeping everything else fixed. Table 3 compares three carriers at $\sigma_{M}=6\;\mu m$ with 200 draws per cell, all scaled to RMS=11.92μm; the bias is measured against the target-set reference of Figure 3 and Figure 4 (full precision in Table S5). Removing the panel-scale content of the measured field lowers the $n_{c}=4$ bias from 84.2% to 50.1%, so it is a real contributor of the size claimed in Section 4.3.5. The ordering does not hold at $n_{c}=8$, where the two variants of the measured field swap places, which is consistent with the control layout being an equally large term: the attribution is firm at $n_{c}=4$ and indicative elsewhere. The noise-free rigid-mismatch metric of Section 4.3.5 gives the same ordering at $n_{c}=4$ (158% for the measured field, 114% after the panel-scale content is removed, 30% for the synthetic carrier).

The bottom line for the acceptance decision is that the CAD-anchored common datum is not hiding error. In the truth convention CD and JDFD sit at +2.5% on the measured field and at −3.6% on the synthetic carrier of Section 4.3.3; on the full-set figure of 11.92μm those correspond to 12.22 and 11.50μm, both of which still exceed the 10μm specification. The verdict is unchanged whichever sign the offset takes, and the offset is an order of magnitude smaller than the 79% that IND(4) reports.

### 4.4. Temporal-Drift Audit of the Zero-Mean Assumption

The decomposition $\hat{I}={\bar{H}}_{i\;\cdot \;}$ relies on the zero-mean assumption $E_{j}\left[M_{ij}\right]=0$. We audit this assumption in two ways: per-epoch similarity parameters and a linear trend in the per-epoch residual RMS.

#### 4.4.1. Per-Epoch Residual Similarity

Table 4 lists the vendor per-epoch CAD fit similarity (machine-readable in Data S4), which characterises the similarity components that JDFD peels. Peak rotation is below 8 arcsec and peak translation below 50 μm; the isotropic scale is 1.0000 to the reported precision in every epoch. The non-zero rotations and translations are precisely the per-epoch similarity components that JDFD removes. The absence of per-epoch scale jitter indicates that scale-bar drift acts as a common-mode term across repeats, which cannot be removed by averaging and therefore remains in $\hat{I}$ rather than in $\hat{M}$ (see Section 5 on common-mode terms). These non-zero similarity components are removed by JDFD; the zero-mean assumption applies to the peeled residual, which is audited next.

#### 4.4.2. Linear Trend

The per-epoch residual RMS $\mathrm{RMS}(H_{\;\cdot \;j})$ shows no significant drift over the five repeats, supporting the zero-mean assumption for $\hat{I}={\bar{H}}_{i\;\cdot \;}$. Student-*t* and Theil–Sen slope CIs both span zero (Table S1). $\mathrm{RMS}(M_{\;\cdot \;j})$ and the mean of $|M_{ij}|$ show a small negative slope of about −0.4μm per repeat index, an order of magnitude below $\bar{\mathrm{RMS}}\left(\hat{M}\right)=3.76\;\mu m$. A purely linear trend in $E_{j}\left[M_{ij}\right]$ averages to zero in $\hat{I}={\bar{H}}_{i\;\cdot \;}$ at first order, so this small slope does not threaten the zero-mean assumption. Full regression output, Student-*t*, Theil–Sen slope CI, Spearman correlation and a Shapiro–Wilk diagnostic are in Table S1.

## 5. Discussion

### 5.1. When JDFD Helps Relative to IND

When the per-epoch datum can be realised from dozens of stiff controls (or the full homologous target set), IND and JDFD give nearly the same $\mathrm{RMS}(\hat{I})$. In that case, the only remaining benefit of JDFD is an uncontaminated $\mathrm{RMS}(\hat{M})$. JDFD is needed when the datum is realised by sparse tooling, when repeats must share one CAD-anchored reference shape, or when $\mathrm{RMS}(\hat{M})$ itself is reported.

On the real five-repeat data (Section 4.3), IND overestimates $\mathrm{RMS}(\hat{I})$ by 79% at $n_{c}=4$—the median over random four-point layouts is ≈51%, and a different tooling layout could have reported almost anything between a few percent and twice the figure—and the gap closes only when $n_{c}\approx 32$. JDFD, in contrast, yields an $\mathrm{RMS}(\hat{I})$ that is independent of the sparse-control budget: it always registers the full mean target set and reproduces the full-set reference at every $n_{c}$ tested. JDFD is also inexpensive: each iteration is one closed-form rigid fit plus *m* independent seven-parameter least-squares peels, so for the present n=7182 targets and m=5 epochs the full decomposition completes in well under a second on a single CPU thread, which makes it practical for routine shop-floor inspection of large assemblies.

### 5.2. Comparison with Simpler Alternatives

The two simpler alternatives each leave one residual unchecked. Full-set IND ($n_{c}=n$) does not strip residual similarity, so $\mathrm{RMS}(\hat{M})$ is inflated by whole-body jitter. $\mathrm{RMS}(\hat{I})$ is unaffected, but the reported repeatability is biased. Sparse-control IND ($n_{c}\ll n$) does not share a common datum, so $\mathrm{RMS}(\hat{I})$ is biased upward when the assembly-to-perturbation ratio is high (Section 4.3.3 and Section 4.3). JDFD is the only estimator tested that achieves both an $n_{c}$-invariant $\mathrm{RMS}(\hat{I})$ and an uncontaminated $\mathrm{RMS}(\hat{M})$. The acceptance decision should therefore rest on $\mathrm{RMS}(\hat{I})$, while $\mathrm{RMS}(\hat{M})$ serves as a secondary quality metric.

### 5.3. Repeatability and Common-Mode Terms

$\mathrm{RMS}(\hat{M})=3.36$ μm is a within-session lower bound on homologous repeatability, and hence a lower bound on the perturbation of routine inspection practice: the panels were not re-adjusted between repeats, and the inter-repeat interval is not characterised, so the figure excludes network re-establishment, operator change, ambient drift and scale-bar handling. It is not day-to-day reproducibility. To show how much this matters for the verdict, suppose the day-to-day terms added an independent per-repeat component of RMS $\sigma_{d}$. The perturbation estimate would rise to $\sqrt{3.36^{2}+\sigma_{d}^{2}}$: 4.51 μm at $\sigma_{d}=3$ μm (+34%) and 6.03 μm at $\sigma_{d}=5$ μm (+79%). The acceptance quantity, however, would move only from 11.92 μm to $\sqrt{\mathrm{RMS}{\left(I^{⋆}\right)}^{2}+\left(\mathrm{RMS}{\left(\hat{M}\right)}^{2}+\sigma_{d}^{2}\right)/m}$, where the residual assembly field is $\mathrm{RMS}(I^{⋆})=11.83$ μm: 12.00 μm and 12.13 μm, respectively, and still only 12.73 μm even at $\sigma_{d}=10$ μm. Because the verdict turns on $\mathrm{RMS}(\hat{I})$, a day-to-day perturbation of several micrometres inflates the reported repeatability substantially while leaving the pass/fail decision unchanged; $\mathrm{RMS}(\hat{M})$ is secondary for acceptance for exactly this reason.

Repeats cannot remove common-mode terms such as a wrong scale bar, an unchanged camera calibration, or an incorrect target thickness. A spatially uniform piston *c* raises $\mathrm{RMS}(\hat{I})$ to $\sqrt{\mathrm{RMS}{\left(\hat{I}\right)}^{2}+c^{2}}$, so it cannot reduce $\mathrm{RMS}(\hat{I})$ below the specification. The same holds for any common-mode field b(x) that is spatially uncorrelated with $\hat{I}$: $\mathrm{RMS}\left(\hat{I}+b\right)=\sqrt{\mathrm{RMS}{\left(\hat{I}\right)}^{2}+\mathrm{RMS}{\left(b\right)}^{2}}$. Only a component that is spatially *antiphase* with the assembly field can lower the RMS, so the verdict would flip from fail to pass only if a common-mode contribution cancelled $\hat{I}$ coherently by a factor α>1−10/11.92=0.16, i.e., by 1.92 μm of coherent antiphase error. A scale-bar, calibration or target-thickness error of the magnitude seen here acts as an approximately uniform or monotone term, and would therefore have to be organised in spatial antiphase with the panel-scale assembly error at the 2 μm level to reverse the decision; the common-datum residuals of Section 4.4 show no such structure. The decision is consequently robust to common-mode terms that raise the RMS, and would require a coherent cancellation comparable to 16% of the assembly field to reverse. Splitting such terms requires an independent instrument on the same nests, for example a laser tracker with a known SMR offset [20,21,22]. Datum definition follows the GPS convention [7,23], but its practical realisation on a panelised assembly requires additional choices about tooling-ball selection and network coverage [4,6].

## 6. Conclusions

JDFD is a restricted GPA for photogrammetric acceptance that shares one CAD-anchored common rigid across repeats and peels per-epoch residual similarity. It yields an assembly RMS that is independent of the sparse-control budget used by IND: at $n_{c}=4$, IND overestimates $\mathrm{RMS}(\hat{I})$ by ≈79% on real data—the median over random four-point layouts is ≈51%, with a 5–95% range of ≈6–168%—and by ≈43% in Monte Carlo ($\sigma_{M}=6.0\;\mu m$), whereas JDFD recovers the full-set reference of 11.92 μm at every $n_{c}$ tested. In addition, peeling residual similarity yields an uncontaminated perturbation $\mathrm{RMS}(\hat{M})=3.36\;\mu m$, compared with 5.57μm without peeling and with the 3.91μm full-set independent-datum baseline. That gain is not reproduced by a Monte Carlo whose epochs carry only white noise (Section 4.3.6); it is specific to the whole-body similarity the delivered networks carry, and it is reported as a measurement rather than as a property of the estimator in general. For the five-repeat inspection of 7182 targets, JDFD gives $\mathrm{RMS}(\hat{I})=11.92\;\mu m$ with a block-bootstrap 95% interval [11.28,12.61]μm, which exceeds the 10μm specification, while $\mathrm{RMS}(\hat{M})=3.36\;\mu m$ represents within-session repeatability under unchanged configuration. These results support using the $n_{c}$-invariant $\mathrm{RMS}(\hat{I})$ as the basis for acceptance decisions.

## Figures and Tables

**Table 1:** Five-repeat decomposition (n=7182). RMS in micrometres. $\mathrm{RMS}(\hat{M})$ is the period-*m* sample RMS of the perturbation field; $\bar{\mathrm{RMS}}\left(\hat{M}\right)=\sqrt{m/(m-1)}\;\mathrm{RMS}\left(\hat{M}\right)$ is the unbiased estimator. **IND** here uses the full target set ($n_{c}=n=7182$).

Quantity	Vendor	IND	CD	JDFD
$\mathrm{RMS}(\hat{I})$	11.90	11.92	11.92	11.92
$\mathrm{RMS}(\hat{M})$	3.67	3.91	5.57	3.36
$\bar{\mathrm{RMS}}\left(\hat{M}\right)$	4.10	4.38	6.22	3.76
Proportion $|{\hat{I}}_{i}|>10\;\mu m$	0.37	0.39	0.39	0.39

**Table 2:** Sparse-control test on the real five-repeat data. $\mathrm{RMS}(\hat{I})$ in micrometres; relative bias is measured against the full-set JDFD reference 11.92μm. In the JDFD rows, “full CD” denotes the common datum estimated from the full target set, “$n_{c}$ init” the per-epoch initialisation from $n_{c}$ controls, and “peel” the residual-similarity peeling.

Estimator	Description	$\mathrm{RMS}(\hat{I})$ (μm)	Relative Bias (%)
IND(4)	per-epoch 6-DoF on $n_{c}=4$	21.34	79.02
IND(8)	per-epoch 6-DoF on $n_{c}=8$	15.65	31.26
IND(16)	per-epoch 6-DoF on $n_{c}=16$	13.59	14.01
IND(32)	per-epoch 6-DoF on $n_{c}=32$	12.15	1.93
IND(64)	per-epoch 6-DoF on $n_{c}=64$	12.00	0.69
IND(128)	per-epoch 6-DoF on $n_{c}=128$	11.99	0.57
IND(7182)	per-epoch 6-DoF on the full target set	11.92	0
JDFD(4)	full CD + $n_{c}$ init + peel	11.92	≤$10^{-5}$
JDFD(32)	full CD + $n_{c}$ init + peel	11.92	≤$10^{-5}$
JDFD(128)	full CD + $n_{c}$ init + peel	11.92	≤$10^{-5}$
JDFD(7182)	full-set JDFD (reference)	11.92	0

**Table 3:** Monte Carlo on three truth fields, $\sigma_{M}=6\;\mu m$, 200 draws per cell. Bias of $\mathrm{RMS}(\hat{I})$ relative to the CD/JDFD target-set estimate on the same draw, in percent. Carrier B is carrier A with its panel-scale content removed (a 400 mm moving average subtracted, the residual rescaled to the same RMS); carrier C is the synthetic paraboloid carrier of Section 4.3.3.

Carrier	*n_c_* = 4	8	16	32	64
A measured field (Data S5)	+84.2	+28.1	+13.7	+2.0	+0.8
B A with panel-scale content removed	+50.1	+41.3	+16.9	+3.2	+0.8
C synthetic 400 mm carrier	+11.3	+30.3	+3.5	+0.7	+0.5

**Table 4:** Per-epoch residual similarity of each vendor CAD fit (zero-mean audit, n=7182). $\mathrm{RMS}(Z_{j})$ is the vendor-reported Z-residual RMS.

Epoch	$\max|\omega_{j}|$ (arcsec)	$∥t_{j}{∥}_{2}$ (μm)	$\sigma_{j}$ (ppm)	$\mathrm{RMS}(Z_{j})$ (μm)
$R_{1}$	0.00	0.0	0	12.0
$R_{2}$	7.20	28.0	0	12.0
$R_{3}$	6.12	46.0	0	12.0
$R_{4}$	5.40	45.0	0	13.0
$R_{5}$	5.40	44.0	0	13.0

## Data Availability

The original contributions presented in this study are included in the article and in the Supplementary Materials (Tables S1–S5, Data S1–S6). In particular, Data S5 (Ihat.csv) provides the estimated per-target assembly field $\hat{I}$ for all n=7182 targets ($\mathrm{RMS}(\hat{I})=11.92\;\mu m$), which is sufficient to reproduce every bias and interval reported here. The raw industrial photogrammetric coordinates of the inspected assembly (the five vendor model-comparison reports) are not publicly available due to proprietary restrictions; Data S5 contains no raw instrument observations. Further inquiries can be directed to the corresponding author.

## Supplementary Materials

The following supporting information can be downloaded at https://www.mdpi.com/article/10.3390/s26216761/s1, Table S1: per-epoch residual similarity and linear-trend audit of the five repeats; Table S2: configuration and cells of the perturbation-model Monte Carlo; Table S3: real-data sparse-control sweep; Table S4: random-control sensitivity summary (200 draws); Table S5: sparse-control Monte Carlo on the measured assembly field with the three-carrier control (Section 4.3.6). Data S1: sys_sim_ctrl_scan.json (perturbation-model Monte Carlo cells, 200 draws per cell); Data S2: real_ctrl_scan_random_nc4.json (200-draw random-control sensitivity: full bias arrays, degenerate counts, percentile summaries and the 95% bootstrap interval on the median); Data S3: real_ctrl_scan.csv/.json (full real-data $n_{c}$ sweep); Data S4: per_epoch_similarity.csv (per-epoch vendor CAD-fit similarity and common-datum residual RMS); Data S5: Ihat.csv (the estimated per-target assembly field $\hat{I}$ for all n=7182 targets, $\mathrm{RMS}(\hat{I})=11.92$ μm), from which every bias reported here can be recomputed independently; Data S6: results_real_field_mc.json (the sparse-control Monte Carlo of Section 4.3.6 with the Data S5 field held fixed as pseudo-truth, its three-carrier control and the noise-free rigid-mismatch diagnostic, seed 20260926).

## Abbreviations

The following abbreviations are used in this manuscript:

CAD	computer-aided design
CD	common-datum estimator
GPA	generalised Procrustes analysis
IND	independent-datum estimator
JDFD	joint datum–form decomposition
RMS	root mean square
6-DoF	six degrees of freedom
